# Mediators of Inflammation and Their Effect on Resident Renal Cells: Implications in Lupus Nephritis

**DOI:** 10.1155/2013/317682

**Published:** 2013-09-22

**Authors:** Susan Yung, Kwok Fan Cheung, Qing Zhang, Tak Mao Chan

**Affiliations:** Department of Medicine, The University of Hong Kong, Hong Kong

## Abstract

Lupus nephritis affects up to 70% of patients with systemic lupus erythematosus and is a major cause of morbidity and mortality. It is characterized by a breakdown of immune tolerance, production of autoantibodies, and deposition of immune complexes within the kidney parenchyma, resulting in local inflammation and subsequent organ damage. To date, numerous mediators of inflammation have been implicated in the development and progression of lupus nephritis, and these include cytokines, chemokines, and glycosaminoglycans. Of these, type I interferons (IFNs) can increase both gene and protein expression of cytokines and chemokines associated with lupus susceptibility, and interleukin-6 (IL-6), tumor necrosis factor-**α** (TNF-**α**) and hyaluronan have been shown to elicit both pro- and anti-inflammatory effects on infiltrating and resident renal cells depending on the status of their microenvironment. Expression of IL-6, TNF-**α**, type I IFNs, and hyaluronan are increased in the kidneys of patients and mice with active lupus nephritis and have been shown to contribute to disease pathogenesis. There is also evidence that despite clinical remission, ongoing inflammatory processes may occur within the glomerular and tubulointerstitial compartments of the kidney, which further promote kidney injury. In this review, we provide an overview of the synthesis and putative roles of IL-6, TNF-**α**, IFN-**α**, and hyaluronan in the pathogenesis of lupus nephritis focusing on their effects on human mesangial cells and proximal renal tubular epithelial cells.

## 1. Introduction

Renal involvement (i.e. lupus nephritis) is a serious manifestation of systemic lupus erythematosus (SLE) that affects up to 70% of SLE patients and is a strong predictor of morbidity and mortality [1]. Depending on the severity of disease, up to 30% of lupus patients will progress to end-stage renal disease and will require dialysis to sustain life. Lupus nephritis is prevalent in non-Caucasian females especially those of child-bearing age and is characterized by a loss of immune tolerance, production of autoantibodies against nuclear antigens and immune-mediated kidney injury [1]. It is initiated by the deposition of immune complexes within the renal parenchyma leading to complement activation, mesangial expansion and induction of inflammatory and fibrotic processes, resulting in glomerulonephritis and progressive renal dysfunction. 

There is evidence to suggest that anti-dsDNA antibodies contribute to the pathogenesis of lupus nephritis since many features of this disease can be reproduced in nonautoimmune mice after intraperitoneal administration of anti-dsDNA antibodies [2]. Anti-dsDNA antibodies are also essential to the diagnosis of SLE, and their levels correlate with disease activity [3, 4]. We and others have demonstrated that anti-dsDNA antibodies can bind to mesangial cells, endothelial cells, and proximal renal tubular epithelial cells to induce cell proliferation, apoptosis, and inflammatory and fibrotic processes [5–14]. The precise mechanisms through which anti-dsDNA antibodies are deposited in the renal parenchyma remain to be defined, but studies suggest that they can bind directly to cross-reactive antigens such as annexin II and *α*-actinin on the surface of mesangial cells or through nucleosomes bound to components of the glomerular basement membrane, where they induce downstream inflammatory processes [10, 15–17]. Histologically, glomerular lesions may range from no or mild mesangial proliferation to highly proliferative and crescentic glomerulonephritis, whereas tubulointerstitial lesions correlate with renal prognosis. Serum levels of IL-6, TNF-*α*, IFN-*α*, and hyaluronan (HA) are increased in patients with lupus nephritis [7, 18, 19]. There is accumulating evidence to demonstrate that their expression is increased in the renal parenchyma of patients and mice with active lupus nephritis, mediated in part through stimulation of resident renal cells with anti-dsDNA antibodies, which contribute to the development and progression of disease (Table 1) [7, 20–25]. Furthermore, their synthesis precedes inflammatory cell infiltration and renal injury. Mesangial cells are an important source of these inflammatory mediators during the early stage of lupus nephritis, but as disease progresses, infiltrating lymphocytes, macrophages, endothelial cells, and proximal renal tubular epithelial cells are activated by IL-6, TNF-*α*, IFN-*α*, and HA, which further drive the inflammatory processes in the kidney and highlight their prominent roles in the pathogenesis of lupus nephritis. This review will discuss the contributing roles of these inflammatory mediators and their synthesis in the pathogenesis of lupus nephritis, with particular focus on their effects in mesangial cells and proximal renal tubular epithelial cells. The contribution of lymphocytes and macrophages in amplifying inflammatory processes during lupus nephritis is outside the scope of this review and has been described elsewhere [26–29].

## 2. Interleukin-6 (IL-6)

IL-6 is a pleiotropic cytokine with an MW of 21 kDa that is secreted by both lymphoid and nonlymphoid cells such as B cells, T cells, monocytes, mesangial cells, proximal renal tubular epithelial cells, endothelial cells, and fibroblasts [10, 30–36]. It is a multifunctional cytokine essential for the differentiation and maturation of B cells, acute-phase protein production, and mesangial cell proliferation. IL-6 can target IFN-inducible genes such as *Ifi202* in murine fibroblasts and splenocytes through activation of STAT3, which results in the suppression of cell cycle progression and inhibition of apoptosis, thereby contributing to increased lupus susceptibility [22]. Serum and urinary IL-6 levels are increased in patients with lupus nephritis, especially in those with diffuse proliferative lupus nephritis, and correlate with nephritic flares [37]. In the normal kidney, IL-6 is localized to the mesangial area and within vascular walls. In patients with lupus nephritis, its expression is increased in mesangial cells, induced in podocytes, and is present in glomerular immune deposits and along the apical aspects of proximal renal tubular epithelial cells [20, 24, 33, 38]. 

The mechanisms through which IL-6 is locally produced in the kidney during pathogenesis of lupus nephritis have not been fully defined. We have recently demonstrated that human polyclonal anti-dsDNA antibodies bind to annexin II on the surface of human mesangial cells and are rapidly internalized to induce downstream inflammatory processes including increased transcription and translation of IL-6, mediated through increased activation of ERK and p38 MAPK [10]. We have also demonstrated that following binding and internalization, the subsequent cellular localization of anti-dsDNA antibodies can influence the amount of IL-6 secreted by mesangial cells. In this respect, induction of IL-6 secretion is more prominent in cells stimulated with anti-dsDNA antibodies with intranuclear localization compared to antibodies that are localized solely to the cytoplasm, and this mechanism of IL-6 induction occurs with autoantibodies derived from patients in remission and with relapse [10]. The importance of anti-dsDNA antibody-annexin II interaction in the induction of IL-6 secretion was corroborated in annexin II gene silencing studies [10]. Induction of IL-6 secretion by anti-dsDNA antibodies has also been observed in rat mesangial cells although the mechanism through which IL-6 was increased was not further investigated [39]. 

The severity of tubulointerstitial lesions is strongly associated with less favorable renal prognosis [40]. Although it was previously believed that glomerular injury provoked tubulointerstitial damage, there is compelling evidence to demonstrate that proximal renal tubular epithelial cells can directly contribute to the pathogenesis of lupus nephritis. Up to 70% of patients with lupus nephritis have discernible immune aggregates and IL-6 expression along the tubular basement membrane [33]. Tubulointerstitial expression of IL-6 correlates with IgG deposition, circulating levels of anti-dsDNA antibodies and tubular abnormalities such as inflammatory cell infiltration, tubular atrophy, and interstitial fibrosis in patients with diffuse proliferative lupus nephritis [33]. Proximal renal tubular epithelial cells constitute the predominant cell type in the tubulointerstitium and play a pivotal role in the immunopathogenesis of various renal parenchymal diseases, acting as an effector of immune-mediated inflammation. Exposure of HK-2 cells, an immortalized proximal renal tubular epithelial cell line [41], with anti-dsDNA antibodies induced *de novo* synthesis of both gene and protein expression of IL-6 [33]. Depending on the disease status, induction of IL-6 secretion in these cells was mediated through distinct mechanisms. We demonstrated that during remission, induction of IL-6 secretion was mediated through the direct actions of anti-dsDNA antibodies or indirectly though the prior stimulation of IL-1*β*. In contrast, anti-dsDNA antibodies isolated from the same cohort of patients during relapse increased IL-6 secretion through prior induction of both IL-1*β* and TNF-*α* secretion, suggesting autoantibody heterogeneity within the same patient during remission and relapse [33]. The ability of anti-dsDNA antibodies obtained from remission patients to induce cytokine production in renal cells in most interesting since it would suggest persistence inflammation, albeit at a lower level to that observed during flare, within the glomerular and tubulointerstitial compartments of the kidney despite clinical quiescence. Given that autoreactive mature naïve B cells are detected in lupus patients during remission, which are precursors of antibody secreting plasma cells [42], it is plausible to suggest that this lymphocyte subset may contribute to persistent autoantibody production and inflammatory processes within the tubulointerstitium during the inactive phase of disease.

Inflammatory processes within the glomerular and tubulointerstitial compartments do not occur in isolation. We have demonstrated that mediators secreted by human mesangial cells and HK-2 cells upon stimulation with anti-dsDNA antibodies can induce IL-6 secretion in the other cell type, suggesting bidirectional communication between the glomerulus and tubulointerstitium. Furthermore, at an identical anti-dsDNA IgG concentration, HK-2 cells demonstrated a more prominent induction of IL-6 secretion compared to mesangial cells, thereby highlighting the importance of proximal renal tubular epithelial cells in the immunopathogenesis of lupus nephritis [33]. Consistent with our findings, immunoglobulins of the IgG subclass isolated from the sera of SLE patients induced IL-6 secretion in proximal renal tubular epithelial cells, which was accompanied by ERK activation [43]. 

Mycophenolic acid (MPA) is the active metabolite of mycophenolate mofetil [44], an immunosuppressive agent used in the treatment of patients with lupus nephritis [45–47]. MPA is a specific inhibitor of lymphocyte proliferation that noncompetitively inhibits inosine monophosphate dehydrogenase, a rate-limiting enzyme that plays a critical role in the *de novo* synthesis of guanosine nucleotides [44]. There is also accumulating evidence to demonstrate that MPA can have a direct effect on non-lymphoid cells and has been shown to inhibit cell proliferation and inflammatory processes in endothelial cells, smooth muscles cells, tubular epithelial cells, fibroblasts, and mesangial cells [9, 48–52]. MPA can suppress matrix protein synthesis in mesangial cells stimulated with exogenous TGF-*β*1 or anti-dsDNA antibodies [9, 53]. We have demonstrated that MPA can suppress anti-dsDNA antibody induction of IL-6 secretion in HK-2 cells, which was accompanied by a reduction in cell proliferation [Ng, Yung and Chan, unpublished data].

The importance of IL-6 in the pathogenesis of lupus nephritis has been highlighted by independent researchers who demonstrated that IL-6 can exacerbate glomerulonephritis and disease manifestations in NZB/W mice, whereas interruption of IL-6 signaling is associated with reduced circulating anti-dsDNA antibody levels, improved renal histology and function, decreased proteinuria, and increased survival in lupus-prone mice [21, 54–57]. Whether disease progression in patients with lupus nephritis can be suppressed by targeting IL-6 remains to be determined.

## 3. Tumour Necrosis Factor-*α* (TNF-*α*)

TNF-*α* is a prototype proinflammatory cytokine that is predominantly synthesized by activated macrophages and lymphocytes, and to a lesser extent by intrinsic renal cells. It is synthesized as a 26 kDa membrane-bound protein that is activated and released as a 17 kDa soluble cytokine by TNF-*α*-converting enzymes belonging to the ADAM (a disintegrin and metalloproteinase) family [58]. Low levels of TNF-*α* mRNA can be detected in lupus-prone mice prior to renal injury [59]. Renal expression and circulating levels of bioactive TNF-*α* are increased during clinical and experimental lupus nephritis and correlate with disease activity [19, 24, 60, 61]. We have previously demonstrated that the induction of TNF-*α* secretion by anti-dsDNA antibodies in mesangial cells and proximal renal tubular epithelial cells was an early event and contributed to increased IL-6 secretion [7, 33]. We further demonstrated that TNF-*α* can act synergistically with anti-dsDNA antibodies obtained from patients with active disease to amplify inflammatory responses, an observation not noted with autoantibodies isolated from patients in remission. Increased TNF-*α* secretion can induce apoptotic cell death in resident renal cells, a mechanism that may initiate organ-specific damage. TNF-*α* may also exert distinct effects on the kidney depending on the immunologic microenvironment during different stages of disease. Administration of TNF-*α* to predisease NZB/W mice delayed the onset of disease, whereas administration of low, but not high, doses of TNF-*α* to lupus-prone mice with active disease exacerbated renal injury [62]. In order to induce renal damage, researchers have suggested that TNF-*α* interacts with pathologic mediators present either locally or in the circulation that are not synthesized in pre-disease lupus-prone mice. Notable, these pathologic mediators have yet to be identified. Higher doses of TNF-*α* may exert a protective effect through the induction of tolerance [62]. In support of this hypothesis, Wallach et al. demonstrated that administration of sublethal doses of TNF-*α* to BALB/c mice resulted in tolerance following TNF-*α* rechallenge [63]. The beneficial role of TNF-*α* in the pathogenesis of disease is substantiated by findings that decreased synthesis of TNF-*α* in NZB/W mice is associated with the development of lupus nephritis [64]. In contrast to these findings, increased intrarenal TNF-*α* expression in NZB/W or MRL/lpr mice correlated with renal inflammation and disease activity, a finding not observed in their congenic littermate [59, 62]. It is possible that genetic predisposition may regulate the effect of TNF-*α* in the pathogenesis of lupus nephritis. So, how should one conclude whether induction of TNF-*α* secretion by anti-dsDNA antibodies in resident renal cells is detrimental or protective? Given that anti-dsDNA antibody-mediated induction of TNF-*α* increased IL-1*β* and IL-6 secretions in mesangial and proximal renal tubular epithelial cells, it is plausible to suggest that under these experimental settings, TNF-*α* exerts a pro-inflammatory effect in the kidney. Induction of TNF-*α* expression in the kidney and cultured proximal renal tubular epithelial cells is mediated in part, through prior activation of p38 MAPK during progressive lupus nephritis [65]. 

Since TNF-*α* exerts dual effects on resident renal cells, administration of agents to block TNF-*α* in SLE patients should be approached with caution. Furthermore, their effectiveness in suppressing disease manifestations remains debatable. Aringer et al. reported that the treatment of patients with lupus nephritis with infliximab, a monoclonal antibody against TNF-*α*, for up to 10 weeks improved proteinuria but also transiently increased anti-dsDNA antibody production, a result of increased apoptotic bodies and thus autoantigens following TNF-*α* depletion [66, 67]. Longer treatment with infliximab was associated with adverse side effects that included fatal pneumonia and brain lymphoma although whether this was attributed to the use of infliximab or prior use of other immunosuppressive agents remains to be determined [66]. In patients with rheumatoid arthritis receiving anti-TNF-*α* therapy, side effects encountered included the development of drug-induced lupus-like syndromes, anti-dsDNA antibody production, and glomerulonephritis [68–70]. How can TNF-*α* mediate disease development? Experimental studies have suggested that TNF-*α* can inhibit type I IFN, a family of pro-inflammatory cytokines known to exert pathogenic roles in the development of lupus nephritis. When TNF-*α* is inhibited by anti-TNF agents, synthesis of type I IFN is no longer repressed, thereby permitting the exacerbation of inflammatory processes. Whether similar findings are observed in lupus patients remain to be determined. The actions of TNF-*α* in the pathogenesis of lupus nephritis have also been confounded by reports that anti-TNF-*α* treatment in NZB/W mice with active lupus nephritis induced by IFN-*α* protected the mice against renal damage and prolonged their survival by attenuating the kidney's response to glomerular immune complex deposition [71]. Blockade of TNF-*α* activity in patients or animals with lupus nephritis may be beneficial or otherwise depending on the dose, treatment duration, and status of disease when treatment is administered. Is it possible to suppress the pro-inflammatory properties of TNF-*α*, while retaining its anti-inflammatory properties?

## 4. Type I Interferons (IFNs)

The type I IFN family consists of IFN-*α*, IFN-*β*, IFN-*ω*, IFN-*κ*, and IFN-*ε*. These pleiotropic cytokines are key regulators of the innate and adaptive immunity, and their levels are increased during antiviral responses and autoimmune diseases [72]. Type I IFNs can promote cell proliferation and differentiation of monocytes and B cells into antigen-presenting cells or plasma cells, respectively. These cytokines mediate their inflammatory responses through their engagement with a common heterodimeric receptor composed of type I IFN receptor 1 and 2 subunits. There is evidence that IFN-*α* plays a critical role in the development of lupus nephritis. Serum levels of IFN-*α* and its expression in the glomeruli of lupus patients correlate with disease activity [73–76]. Studies have suggested that increased serum IFN-*α* bioactivity and polymorphism of interferon regulatory factor 5 gene, a transcription factor essential for IFN-*α* secretion, are associated with SLE and lupus nephritis susceptibility, respectively [77, 78]. Patients with viral infections or malignant tumors frequently develop SLE-like manifestations and anti-DNA antibodies following IFN-*α* treatment, thereby corroborating the importance of this cytokine in the development of lupus [79–81]. Although plasmacytoid dendritic cells are the primary source of type I IFNs in lupus patients, intrinsic renal cells such as mesangial cells and glomerular endothelial cells can also synthesize IFN-*α* following stimulation with viral components mediated through toll-like receptor dependent and independent pathways [82–84]. Synthesis of IFN-*α* by endothelial cells may contribute to the infiltration of inflammatory cells into the kidney parenchyma. Stimulation of mesangial and proximal renal tubular epithelial cells with anti-dsDNA does not induce IFN-*α* secretion (Yung and Chan, unpublished observation). Fairhurst et al. demonstrated that type I IFNs synthesized by resident renal cells in an experimental model of anti-GBM nephritis induced renal dysfunction, glomerulonephritis, crescent formation, and tubulointerstitial nephritis [85]. 

Exposure of NZB/W mice to IFN-*α* can accelerate pathogenic autoantibody production, proteinuria development, and glomerular IgG deposition and render these mice more resistantly to therapeutic intervention when compared to lupus-prone mice without IFN-*α* treatment [23, 86, 87]. Gene silencing of IFN-*α*/*β*R in NZB mice, which ablated the biological activities of IFN-*α*/*β*, suppressed splenomegaly, anti-dsDNA antibody production, and kidney pathology and improved survival compared to their wildtype littermates, thereby substantiating the pathogenic role of type I IFN in promoting SLE [88]. 

IFN-induced mRNA transcripts, otherwise known as IFN-signature, are increased in peripheral blood mononuclear cells isolated from SLE patients and may serve as a marker for more severe organ manifestations such as lupus nephritis [89, 90]. Increased IFN-*α*-inducible transcripts have been observed in the glomeruli of patients with lupus nephritis, which inversely correlated with many genes that promote renal fibrosis [91]. Intriguingly, this would suggest that the expression of IFN-inducible transcripts could either result in a milder form of renal injury or be protective against glomerular damage [91]. Whether the presence of this signature is a cause or consequence of disease remains to be fully defined. 

## 5. Hyaluronan (HA)

HA is a large, negatively-charged, nonsulfated glycosaminoglycan composed of repeating disaccharide units of D-glucuronic acid and N-acetyl-D-glucosamine [92]. It is synthesized on the inner surface of the plasma membrane by HA synthases (HAS), and newly synthesized HA is either directed to the cell surface where it interacts with its receptor CD44 or is assembled into the extracellular matrix. Three HAS isoenzymes have been identified that share 55–70% homology and are termed HAS I, HAS II, and HAS III [93]. Under physiologic conditions, HA may possess up to 25,000 disaccharide units with a corresponding molecular mass of 10^6^–10^7^ Da [94]. HA contributes to basement membrane stability and sequestration of free radicals and plays critical roles in cell proliferation, differentiation, migration, and phenotypic changes. 

In the normal kidney, HA is primarily expressed within the inner medulla interstitium where it contributes to the mechanical stability of tubules and blood vessels, and also in the concentration of urine [95]. In patients and mice with active lupus nephritis, HA expression extends into the renal cortex and has a periglomerular, crescentic, mesangial, and tubulointerstitial distribution [7, 96, 97]. We and others have demonstrated that cultured mesangial cells, glomerular endothelial cells, proximal renal tubular epithelial cells, and interstitial fibroblasts can synthesize HA [7, 98–103], and it is thus plausible to suggest that these cell types contribute to increased HA levels in the renal cortex in lupus patients. HA accumulation in the tubulointerstitium correlates with lymphocyte infiltration and renal damage mediated in part, through prior induction of TNF-*α* and IFN-*γ* [97]. There is mounting evidence to suggest that during chronic kidney inflammation, mesangial cells and proximal renal tubular epithelial cells synthesize HA that forms long cable-like structures that function as an adhesive matrix, which binds leukocytes and macrophages preventing them from interacting with adhesion molecules, thereby limiting glomerular and tubulointerstitial inflammation [104–106]. 

Human polyclonal anti-dsDNA antibodies can increase high molecular weight (HMW) HA synthesis and induce synthesis of low molecular weight (LMW) HA in mesangial and proximal renal tubular epithelial cells when compared to control cells, mediated in part through prior induction of IL-1*β* secretion, and increased HAS II gene expression [7, 107]. Independent researchers have demonstrated that the biological functions of HA is governed by its molecular weight. HMW HA possesses anti-inflammatory and antiangiogenic properties and can promote cell quiescence, whereas LMW HA is pro-inflammatory and can induce cytokine and chemokine secretion, activation of signalling pathways, cell proliferation, and angiogenesis [108–113]. The presence of LMW HA can arise through *de novo* synthesis during inflammation or through the depolymerisation of native HA following increased hyaluronidase activity or exposure to reactive oxygen species [110]. Pro-inflammatory cytokines can increase synthesis of both HMW and LMW HA in various cell types. Although we and others have shown an increase in intrarenal HA expression in patients and mice with active lupus nephritis, its MW was not investigated [7, 97, 114]. It is noteworthy that despite differences in the biological functions of HMW and LMW HA, reports detailing the presence of LMW HA in tissues undergoing inflammation and injury are scarce. 

Exogenous LMW, but not HMW HA, can induce MCP-1 mRNA and protein secretion in proximal renal tubular epithelial cells [25]. Glomerular and tubulointerstitial expression of MCP-1 is increased in lupus-prone mice and precedes leukocyte infiltration, proteinuria, and renal damage [115]. The significance of MCP-1 in the pathogenesis of lupus nephritis was highlighted by Tesch et al. who observed that MRL/lpr mice deficient in MCP-1 demonstrated increased survival consequent to less severe renal histology and proteinuria compared to their wildtype littermates [116]. We have demonstrated that suppression of HA synthesis in NZBWF1/J mice was associated with an improvement in clinical parameters of disease and decreased intrarenal expression of IL-6 and TNF-*α* [96]. It would be most interesting to determine whether HA is a potential target for therapeutic intervention in the pathogenesis of lupus nephritis.

Serum HA levels are increased in patients with lupus nephritis compared to healthy controls and correlate with circulating anti-dsDNA antibody levels, suggesting that these autoantibodies contribute to increased HA synthesis [7]. It is noteworthy that an increase in the level of circulating HA is not specific to lupus but rather, a consequent of renal impairment and injury [117–119]. Elevated serum HA levels are associated with inflammation, malnutrition, and poor prognosis in patients with end-stage renal failure [120].

## 6. Conclusions

Although a plethora of inflammatory mediators have been implicated in the development and pathogenesis of lupus nephritis, our understanding of how they mediate renal injury has been confounded by their multifunctional roles. There is compelling evidence to demonstrate that resident renal cells can directly contribute to renal inflammation through their ability to secrete cytokines, chemokines, and glycosaminoglycans following their interaction with, but not limited to, anti-dsDNA antibodies and viral components.

Disease remission and prevention of irreversible renal damage are the ultimate goals of induction therapy but irrespective of achieving clinical remission, studies have demonstrated that low-grade inflammatory processes may persist within the renal parenchyma leading to further kidney injury. Further research into the mechanisms through which pro-inflammatory mediators are modulated during disease manifestations, how they interact with other inflammatory mediators, and the underlying mechanisms that dictate whether these molecules elicits pro- or anti-inflammatory responses will provide us with a better understanding of their roles in lupus nephritis and whether targeting these molecules can improve clinical outcome without affecting their physiological roles.

## Figures and Tables

**Table 1:** Mediators of inflammation that play important roles in the pathogenesis of lupus nephritis.

Inflammatory mediator	Putative roles in lupus nephritis
IL-6	(i) Activates B cells
	(ii) Induces glomerulonephritis
IFN-*α*	(i) Interferes with vascular repair by inducing endothelial progenitor cell apoptosis
	(ii) Induces renal dysfunction, glomerulonephritis, crescent formation, and tubulointerstitial nephritis
IFN-*γ*	Promotes macrophage recruitment into the kidney and the development of glomerulonephritis
TNF-*α*	(i) Regulates physiological and inflammatory immune responses
	(ii) Induces synthesis of IL-1*β* and IL-6 in mesangial cells and proximal renal tubular epithelial cells
	(iii) Elicits both proinflammatory and anti-inflammatory actions in lupus nephritis
Hyaluronan (HA)	(i) Forms HA cables that can prevent leukocyte adhesion to their receptors
	(ii) Induces chemokine secretion
	(iii) Possesses proinflammatory and anti-inflammatory properties
